# Association between congenital toxoplasmosis and parent-reported developmental outcomes, concerns, and impairments, in 3 year old children

**DOI:** 10.1186/1471-2431-5-23

**Published:** 2005-07-13

**Authors:** Katherine Freeman, Alison Salt, Andrea Prusa, Gunilla Malm, Nicole Ferret, Wilma Buffolano, Dorthe Schmidt, Hooi Kuan Tan, Ruth E Gilbert, The European Multicentre Study on Congenital Toxoplasmosis (EMSCOT)

**Affiliations:** 1 Albert Einstein College of Medicine, Department of Epidemiology and Population Health, New York, U.S.A; 2 The Neurodisability Service, Great Ormond Street Hospital for Children and Institute of Child Health, London, UK; 3 Department of Pediatrics, Division of Neonatology and Intensive Care, Medical University of Vienna, Austria; 4 Karolinska University Hospital, Huddinge, Stockholm, Sweden; 5 CHU de NICE, Service Parasitologie – Mycologie, Hopital L'Archet II, BP 3079, 06202 NICE Cedex 3, France; 6 Perinatal Infection Unit, Dept of Pediatrics, University of Naples Federico II, Naples, Italy; 7 Department of Parasitology, Staten Seruminstitut, Copenhagen, Denmark; 8 Centre for Paediatric Epidemiology and Biostatistics, Institute of Child Health, London, UK

## Abstract

**Background:**

Information is lacking on the effects of congenital toxoplasmosis on development, behavior, and impairment in later childhood, as well as on parental concerns and anxiety. This information is important for counselling parents about the prognosis for an infected child and for policy decisions on screening.

**Methods:**

We prospectively studied a cohort of children identified by screening for toxoplasmosis in pregnant women or neonates between 1996 and 2000 in ten European centers. At 3 years of age, parents of children with and without congenital toxoplasmosis were surveyed about their child's development, behavior, and impairment, and about parental concerns and anxiety, using a postal questionnaire.

**Results:**

Parents of 178/223 (80%) infected, and 527/821 (64%) uninfected children responded. We found no evidence that impaired development or behavior were more common in infected children, or that any potential effect of congenital toxoplasmosis was masked by prenatal treatment. Parents of infected children were significantly more anxious and reported more visual problems in their children.

**Conclusion:**

On average, children aged three to four years with congenital toxoplasmosis identified by screening and treated during infancy in this European setting had risks of abnormal development and behavior similar to uninfected children. Parental anxiety about infected children needs to be addressed by clinicians. Future studies with longer follow up and clinician-administered assessments may be better able to detect any subtle differences in child outcomes.

## Background

Congenital toxoplasmosis is associated with a wide spectrum of clinical signs and symptoms. At its most severe, congenital toxoplasmosis causes death or severe disability in 1–4% of infants identified by prenatal or neonatal screening[1]. Although the remaining 96% of infected infants appear clinically normal in infancy, one in six have intracranial and/or ocular lesions. Clinicians lack information for counselling parents about their child's functional abilities in later childhood, and whether intracranial or ocular lesions predict adverse functional outcomes. In addition, policy makers need to know what proportion of children with congenital toxoplasmosis suffer long term functional impairment. However, a systematic search of the literature found only one study of children identified by screening in which school performance in 11 infected children was compared with their peers at 7 years[2]. No difference was found but this may be due to the sample size and/or the insensitivity of the outcome measure. Other studies of developmental outcomes have been based on case series of referred and usually symptomatic children with congenital toxoplasmosis, and have not included an appropriate comparison group[3,4].

We wanted to know whether 3-year-old children with congenital toxoplasmosis are more at risk of adverse developmental or behavioral outcomes than uninfected children. We also investigated parental concerns and anxiety as clinicians caring for infected children highlighted parental anxiety as a common clinical problem. We conducted a prospective cohort study of children identified by prenatal or neonatal screening for toxoplasmosis. Infected and uninfected children born to infected women were followed up during infancy and then surveyed using a parent-completed questionnaire to assess development and other outcomes when the child turned 3 years. This design aimed to ensure that, apart from congenital infection status, the experience of screening, and follow up in early infancy, was similar. However, only infected children received prolonged postnatal treatment and follow up.

## Methods

### Study population

We compared children, with and without congenital toxoplasmosis, born to women identified by prenatal screening for maternal toxoplasmosis between 1996 and 2000 in eight centers (Lyon, Paris, Marseille, Toulouse, Nice, Grenoble, Vienna, and Naples), and by neonatal screening in two centers (Stockholm, Poznan). One other center, Denmark, was excluded from the analyses as no uninfected children were recruited. Details of the methods have been reported elsewhere [5,6]. In brief, 91% of the women in the prenatal screening centers[6], but none in the neonatal screening centers, received anti-toxoplasma treatment before birth. In Poznan (Poland), children were identified by universal neonatal screening for specific IgM in filter paper blood spots from the Guthrie card and uninfected controls were selected as the next six children with a negative screening test born after each infected child[5].

### Clinical follow up

Women suspected to have acquired toxoplasma infection during pregnancy and infected infants identified by neonatal screening were enrolled prospectively, prior to the collection of follow up data. We used a standard questionnaire to record clinical findings during pregnancy, at pediatric examinations in the neonatal period, at six and 12 months, and at ophthalmoscopy before four months and at 12 months of age. Cranial ultrasound was performed within the first four months of life. The number of examinations (pediatric, ophthalmic, and cranial ultrasound) was similar in infected and uninfected children up to 4 months of age.

Confirmation of congenital infection status was based on the persistence of IgG antibodies after 11.5 months of age (infected), or undetectable specific IgG antibodies in the absence of treatment, which usually occurred between 8 and 12 months[6]. To avoid potential biases due to exclusion of 15% of infants who did not meet these confirmatory criteria, we used probability estimations of their congenital infection status based on PCR analysis of amniotic fluid, specific IgM or IgA in the infant, last available IgG results, and the weeks of gestation at maternal seroconversion[6]. All infected children received treatment from early infancy for 12 to 24 months, depending on the centre, based on the results of prenatal diagnosis, and/or a positive IgM/IgA test, or a lack of decline in specific IgG titers. The median age at the start of postnatal treatment in prenatal centers was 2 days (IQR: 0, 14), and in neonatal screening centers 26 days (IQR: 22, 33). Few uninfected children were treated postnatally. As prenatal treatment after fetal diagnosis, and postnatal treatment, were given predominantly to those with evidence of congenital infection, postnatal treatment could not be investigated in this analysis. In Poznan, the uninfected children had no involvement in the cohort until their parents were sent a questionnaire when they turned 3 years. As no clinical follow up information was available for these children, they are excluded from analyses of children with no signs or symptoms in infancy.

We included all children identified by prenatal or neonatal screening in the parent-questionnaire study at 3 years, with some exceptions. First, in Austria and Italy, we randomly selected 4 uninfected children for each infected child, stratified by year of birth, to avoid surveying approximately 9 uninfected children for every infected child in these centers. Second, to minimize potential selection biases, we excluded children with non-sequential dates for the detection of maternal infection, fetal infection or abnormality, or, in the neonatal centre, for screening and confirmatory tests, as these women or children may have been referred. Third, 21 infected children known to be lost to follow up were not surveyed (4 died, but no serious outcomes were documented by paediatricians in the remainder).[7] Fourth, two centers participating in the initial cohort[6] did not participate in the 3 year follow up (Reims, Milan), and one did not enroll uninfected controls (Copenhagen). A detailed description and evaluation of questionnaire response has been reported elsewhere.[7] In brief, the questionnaire was composed of separate assessment tools (in total or in part) for behavior, speech and language, cognition, and motor skills, that have been previously validated against clinician assessments[7]. To-date the questionnaire as a whole has not been validated with standardized clinician assessments. The postal questionnaire, with a stamped addressed envelope for reply, and crayons for the child, was sent to parents by the local study centre when the child turned 3 years. Non-responders were sent two reminders at 2-monthly intervals. Research Ethics approval was obtained for all participating centres.

### Outcomes

The results for the effect of congenital toxoplasmosis and potential confounders are presented for three groups of outcomes. The first comprised developmental outcomes (gross motor, speech and language, and cognitive), and abnormal behavior. These were measured by questions derived from standardized tools that had been validated against clinician assessments[7]. The questionnaire also included two child-completed sections that assessed cognitive and fine motor development. Children were asked to copy a line, circle and cross, drawn by their parent, and secondly, to draw a man[8]. Child-completed measures were analyzed separately from parent-completed measures because not all children participated, and we could not standardize the degree of assistance given to the child.

The second group of outcomes measured parental concerns about development, learning, behavior, and speech and language, specialist referral, and asked parents to rate how worried they were about their child's general health at the time and in the future[9]. Parental concerns about specific areas of development, or specialist referral may be proxy markers of abnormal development[10,11]. More general anxiety about the child's health now and in the future may reflect anxiety generated by diagnosis, treatment and follow up[9], but may also be a marker for abnormality. The third group of outcomes comprised specific impairments that parents were asked about, including difficulty hearing or seeing, and whether the child had cerebral palsy or seizures. The primary outcomes were the scores for motor development, speech and language, cognition, and behaviour, and parental anxiety.

### Analyses

We aimed to compare infected and uninfected children, stratified by centre because of differences in screening programs and treatment regimens, and possible differences in expectations and attitudes to congenital toxoplasmosis. There was also significant variation among centers for exposure and outcome variables. We therefore present the preliminary bivariate analyses to show the effect of centre on outcomes, and adjust all subsequent analyses for center, nested within country, and congenital infection status. Stockholm and Naples were grouped as one centre, due to small numbers. Outcome variables were defined by a score measured on an interval scale (further details reported elsewhere[6]. Abnormality was defined by a cut-point approximating the least able 10% of controls, or for behavior, using an established cut point for abnormality (equivalent to 10% in a large community sample in which the questionnaire was validated[12]). Missing data for mother's age was imputed using a procedure for both continuous and categorical variables[13]

Multivariate analyses of the effect of congenital toxoplasmosis included centre nested within country, maternal education, and child's age at questionnaire completion, in every model based on evidence of confounding in some comparisons and consensus among investigators as to their clinical relevance. We included other potential confounders with p values = 0.20 from the bivariate analyses[14], but retained those variables with p-values <0.05 in the final model. Where the effect of congenital toxoplasmosis was significant (= 0.05), we tested for an interaction between centre and infection status. We used a hierarchical generalized linear model to account for heterogeneity among centres within France and between centres inside and outside France[15] A generalized estimating equation (SAS Version 9.1 PROC GENMOD with the ASSESS options to assess fit of the model) with centers nested within country was derived to determine characteristics associated with each dichotomized outcome. Odds ratios were derived by exponentiating parameter estimates, as well as lower and upper bounds for corresponding 95% confidence intervals. For logistic regression, Wald estimates were used.

We performed several sensitivity analyses for the association between congenital toxoplasmosis and development and behavior, and parental concerns and anxiety, and specialist referral. First, to avoid spurious associations due to dichotomization of outcomes, we repeated the main analyses using ordinal logistic regression for all outcomes measured using an ordinal scale (motor, speech and language, and cognitive development, behavior, child completed drawings, impact of behavior on family, and parental anxiety). Second, for the main model with dichotomized outcomes primary analyses using country as a fixed effect, were repeated using a generalized mixed model approach with country as a random effect Second, to test whether the effect of congenital toxoplasmosis on outcomes differed across countries, we derived separate multivariate models for each country. Third, to minimize the confounding effect of child's age at questionnaire completion on developmental outcomes, we restricted analyses to children aged 36 to 40 months. Fourth, to minimize confounding due to clinical manifestations detected in early infancy, we restricted analyses to children who had no clinical signs identified by the first 4 months of life. Signs were defined as intracranial calcification or ventricular dilatation on ultrasound, retinochoroiditis, lymphadenopathy or hepatosplenomegaly, microphthalmia, microcephaly, seizures, or an abnormal neurological examination. These analyses excluded Poland, where uninfected children were not assessed by the study during infancy. Fifth, to determine whether the duration of prenatal treatment may have masked an effect of congenital toxoplasmosis, we analyzed the effect of congenital toxoplasmosis, adjusted for prenatal treatment duration, and gestational age at maternal seroconversion, in French women who seroconverted during pregnancy. In these women, the gestational age at seroconversion could be estimated fairly precisely based on the midpoint between a negative and positive specific IgM test (usually a one month interval), or 14 days before a positive specific IgM result and negative IgG result[16]. Finally, to detect any adverse effects of prenatal treatment, we confined analyses to uninfected children, using the same approach as for the main model.

Without taking into account adjustment for confounders, the study was designed to have 80% power to detect a minimum difference of 0.22 standard deviations, assuming 70% response in the controls, 80% response in infected children, and an alpha error of 0.05.

## Results

### Study population

Of the 1044 families sent a questionnaire in 10 centers, parents of 178/223 (80%) infected, and 527/821 (64%) uninfected children responded. The distribution of infected and uninfected children by centre was: Lyon (40, 94), Paris (41,50), Marseille (18, 51), Nice (5, 28), Toulouse (18, 26), Vienna (18, 116), Stockholm (2, 6), Naples (10, 40), Poznan (23, 111). Details about factors associated with response, reasons for non-response, and exclusions due to death or potential referral bias, are reported elsewhere^7^. Most parents completed the entire questionnaire, as shown by the denominator for each outcome in Table 1. However, far fewer children completed the drawings: 70% (493/705) completed the draw a man test; and 95% (670/705) copied a line, circle or cross. Few parents reported neurological problems or impaired mobility (N = 6), but visual impairment (n = 10), and hearing loss was more common (N = 37).

**Table 1:** Number of children with an adverse outcome according to congenital infection status (%)

**1) DEVELOPMENT AND BEHAVIOUR**					
	PARENT COMPLETED				
**Infection status**	Motor^1^	Speech & language^1^	Behaviour^2^	Cognition^1^	
CT+ (n = 178)	24/178 (13.5)	20/177 (11.3)	34/176 (19.3)	9/178 (5.1)	
CT- (n = 527)	58/525 (11.0)	50/524 (9.5)	97/527 (18.4)	31/525 (5.9)	
	CHILD COMPLETED				
**Infection status**	'Draw a Man'^1^	Line, circle, cross^1^			
CT+ (n = 178)	17/121(14.0)	13/170(7.6)			
CT- (n = 527)	54/372(14.5)	38/500(7.4)			
**2) PARENTAL CONCERNS, SPECIALIST REFERRAL, AND PARENTAL ANXIETY**					
	CONCERNS				
	Learning/ development^3^	Speech & language^3^	ANY^3^		
CT+ (n = 178)	24/176(13.6)	16/177(9.0)	34/178(19.1)		
CT- (n = 527)	53/522(10.2)	55/522(10.5)	79/527(15.0)		
	REFERRALS				
	Learning/ development	Speech & language	ANY	Behaviour impact ^2^	Parental anxiety^1^
CT+ (n = 178)	10/178(5.6)	4/178(2.3)	11/178(6.2)	3/173 (1.7)	41/176 (23.3)
CT- (n = 527)	13/527(2.5)	14/527(2.7)	20/527(3.8)	16/509 (3.1)	61/521 (11.7)
**3) PARENT-REPORTED IMPAIRMENT**					
	VISUAL IMPAIRMENT				
	Glasses only	Strabismus/ blind/limited vision	ANY	Hearing loss^4^	Neurological/mobility impairment^5^
CT+ (n = 178)	5	10	15/177 (8.5)	7/166 (4.2)	2/175 (1.1)
CT- (n = 527)	11	13	24/527 (4.6)	30/498(6.0)	4/522 (0.8)

Full questionnaire available from the EMSCOT website [22]

CT = congenital toxoplasmosis

^1^Adverse outcome defined by score corresponding most closely to the least able/most worried 10% in the uninfected children

^2^Adverse outcome defined by developers of SDQ questionnaire. Equivalent to lowest 10% in community sample of 10,000 examined in evaluation study. [12]

^3^Defined by answering 'yes' or 'a little' to question(s) about concerns.

^4^Parents reported intermittent or permanent hearing loss

^5^Parents reported seizures requiring treatment, cerebral palsy, and/or impaired mobility

### Potential confounders

The results for the associations between exposures and the three groups of outcomes are shown in Tables 2,3 and 4, adjusted for center and congenital infection status. Maternal age and education level, and child's age at questionnaire completion had significant effects on parent-reported developmental outcomes (Table 2), but not on parental concerns and anxiety (Table 3), or specific impairments (Table 4). Adverse cognitive development was reported more commonly in Grenoble compared with Lyon, and behavior problems were reported more commonly in Marseille. There were significant associations between congenital infection status and parental anxiety (Table 3). Parents in Poznan, reported more anxiety than in Lyon, and children in Poznan, and Stockholm/Naples were more likely to be referred to a specialist. There were more parental concerns about children in Poznan than in Lyon, and about girls than boys. Table 4 shows that visual impairment was more common in infected children, and was reduced in children with longer duration of prenatal treatment. Hearing loss appeared to be less common with increasing parity.

**Table 2:** Factors associated with development and behaviour at 3 years, adjusted for centre and congenital infection status (Total N = 705)

**EXPOSURES**	**Infection status**		**Odds ratio for abnormal developmental outcome **(95% confidence interval)					
				**Parent completed**			**Child completed**	
***Before birth***	**CT+**	**CT-**	Motor	Speech & language	Behaviour	Cognition	'Draw a Man'	Line, circle, cross
**1) Congenital toxoplasmosis **(reference = uninfected)	178	525	1.35 (0.79, 2.30)	1.32 (0.75, 2.33)	1.10 (0.70, 1.73)	0.88 (0.37, 2.08)	0.94 (0.51, 1.74)	1.05 (0.53, 2.07)
**2) Maternal age (yrs) **^1^*								
<25	40	86	**1.07** **(1.02, 1.12)**	**0.93** **(0.88, 0.99)**	**0.92** **(0.87, 0.97)**	0.97 (0.90, 1.05)	1.04 (0.99, 1.09)	1.00 (0.93, 1.07)
25–35	106	288						
>35	27	35						
**3) Parity**^1^*								
0	40	179	1.00 (0.80, 1.24)	1.18 (0.94, 1.48)	0.97 (0.74, 1.27)	1.22 (0.93, 1.60)	0.96 (0.70, 1.31)	1.02 (0.74, 1.40)
= 1	72	212						
Missing	66	136						
**4) Maternal education**^1^								
Primary	14	48	1.15 (0.89, 1.48)	**0.58** **(0.44, 0.77)**	**0.77** **(0.63, 0.94)**	0.74 (0.52, 1.05)	1.20 (0.87, 1.66)	1.01 (0.73, 1.38)
Secondary Lower	47	181						
Secondary Upper	47	135						
Further Education	66	152						
Missing	4	11						
**5) Mother born outside country**^2^								
Yes	25	60	1.45 (0.74, 2.84)	**2.30** **(1.14, 4.63)**	1.65 (0.92, 2.96)	2.25 (0.88, 5.78)	0.89 (0.39, 2.01)	1.66 (0.72, 3.83)
no (reference)	152	462						
Missing	1	5						
**6) Child's gender**^2^								
Male (reference)	95	284	1.45 (0.74, 2.84)	0.81 (0.49, 1.36)	1.65 (0.92, 2.96)	2.25 (0.88, 5.78)	0.89 (0.39, 2.01)	1.66 (0.72, 3.83)
Female	83	232						
Missing	0	11						
**7) Duration of prenatal treatment**^1 ^(mean wks, sd)	6.3 (6.5)	15.1 (12.2)	1.00 (0.98, 1.02)	1.02 (0.99, 1.05)	1.00 (0.98, 1.02)	0.99 (0.96, 1.02)	0.98 (0.96, 1.01)	1.00 (0.98, 1.03)
Missing	0	0						
***Exposures after birth***								
**8) Child's age at questionnaire**^1 ^(months) Median, IQR	38 (37,40)	39 (38, 41)	**0.81** **(0.71, 0.93)**	**0.87** **(0.77, 0.98)**	0.98 (0.93, 1.04)	**0.73** **(0.56, 0.97)**	**0.88** **(0.80, 0.97)**	**0.73** **(0.60, 0.88)**
Missing	0	1						

^1^Odds ratio expresses risk of adverse developmental/behavioural outcome per unit increase in exposure, adjusted for centre and congenital infection status.

^2^Odds ratio expresses risk of adverse developmental/behavioural outcome, adjusted for centre and congenital infection status

**Table 3:** Factors associated with parental concerns, specialist referral, impact of behaviour on the family, and parental anxiety, at 3 years, adjusted for centre and congenital infection status(Total N = 705)

	**Odds ratio for outcome **(95% confidence interval)			
**EXPOSURES**	Any concerns^5^	Any referral	Behaviour impact^4^	Parental anxiety^3^
***Before birth***				
**1) Congenital toxoplasmosis **(reference = uninfected)	0.98 (0.95, 1.01)	2.00 (0.90, 4.45)	0.55 (0.22, 1.39)	**3.01 (1.84, 4.94)**
**2) Maternal Age**^1^	0.97 (0.92, 1.03)	0.96 (0.89, 1.05)	0.98 (0.90, 1.07)	0.98 (0.93, 1.03)
**3) Parity**^1^	0.96 (0.81, 1.15)	1.05 (0.72, 1.54)	0.98 (0.66, 1.44)	1.26 (1.06, 1.50)
**4) Maternal Education**^1^	0.91 (0.73, 1.14)	0.77 (0.51, 1.16)	1.18 (0.81, 1.74)	0.82 (0.65, 1.04)
**5) Mother born outside country**^2 ^(no = reference)	1.59 (0.84, 3.01)	0.82 (0.18, 3.79)	2.20 (0.86, 5.60)	1.06 (0.51, 2.21)
**6) Child's gender **(male = reference)	**1.89 (1.22, 2.93)**	0.83 (0.39, 1.73)	0.99 (0.50, 1.96)	1.26 (0.81, 1.94)
**7) Duration of prenatal treatment **(wks)	0.98 (0.96, 1.00)	1.00 (0.96, 1.04)	1.01 (0.97, 1.05)	**0.97 (0.94, 0.99)**
*After birth*				
**8) Child's age at Questionnaire**^1 ^(month)	0.97 (0.90, 1.04)	1.07 (0.97, 1.19)	1.02 (0.93, 1.13)	0.97 (0.90, 1.04)

^1^Odds ratio expresses risk of adverse developmental/behavioural outcome per unit increase in exposure, adjusted for centre and congenital infection status

^2^Odds ratio expresses risk of adverse developmental/behavioural outcome, adjusted for centre and congenital infection status

^3^Adverse outcome defined by score corresponding most closely to the least able/most worried 10% in the uninfected children

^4^Adverse outcome defined by developers of SDQ questionnaire.

^5^Defined by answering 'yes' or 'a little' to question(s) about concerns.

**Table 4:** Factors associated with parent-reported impairment at 3 years adjusted for centre and congenital infection status (Total N = 705)

	**Odds ratio for adverse outcome **(95% confidence interval)		
**EXPOSURES**	Any visual impairment^3^	Hearing loss^4^	Neurological/mobility impairment^5^
*Before birth*			
**1) Congenital toxoplasmosis**	**2.22 (1.10, 4.49)**	0.61 (0.26, 1.46)	1.88 (0.32, 11.04)
**2) Maternal Age**^1^	0.99 (0.92, 1.07)	0.91 (0.92, 1.07)	1.15 (0.94, 1.42)
**3) Parity**^1^	1.08 (0.82, 1.43)	**0.77 (0.84, 0.99)**	1.63 (1.01, 2.63)
**4) Maternal Education**^1^	1.13 (0.77, 1.65)	0.96 (0.67, 1.37)	0.70 (0.27, 1.79)
**5) Mother born outside country**^2 ^(no = reference)	0.35 (0.08, 1.53)	0.54 (0.16, 1.87)	Not estimable
**6) Child's gender **(male = reference)	1.76 (0.88, 3.51)	1.27 (0.64, 2.50)	0.78 (0.15, 3.98)
**7) Duration of prenatal treatment **(wks)	**0.95 (0.92, 0.99)**	1.01 (0.97, 1.04)	0.96 (0.87, 1.06)
*After birth*			
**8) Child's age at Questionnaire**^1 ^(month)	1.02 (0.93, 1.11)	0.90 (0.79, 1.03)	1.05 (0.86, 1.29)

^1^Odds ratio for effect of exposure on outcome in all subjects per additional unit increase in exposure variable, adjusted for centre ^2^Odds ratio for effect of exposure on outcome, adjusted for centre ^3^Defined as any visual impairment, including wearing glasses ^4^Defined as any intermittent or permanent hearing loss ^5^Defined as seizures requiring treatment, cerebral palsy, and/or impaired

Information on clinical signs before four months of age was available for 571 children (excluding 23 infected and 111 uninfected children in Poznan). 131/155 (85%) infected children and 407/414 (98%) uninfected children had no clinical signs. In the remainder, clinical manifestations were detected before four months of age in order of increasing severity: lymphadenopathy or hepatosplenomegaly (3 infected, 2 uninfected); retinochoroiditis alone (6 infected, 0 uninfected); intracranial lesions, with or without retinochoroiditis (13 infected, 3 uninfected); and neurological impairment, with or without ocular or intracranial lesions and including seizures, microcephaly, microphthalmia, or abnormal neurological examination (2 infected, 4 uninfected).

### Multivariable analyses

#### Development and behavior

There was no significant association between congenital infection status and abnormal development or behavior (Table 5). Similar results were obtained in the sensitivity analyses. In analyses confined to France, there was no evidence that an adverse effect of congenital infection status was masked by the duration of prenatal treatment, or that prenatal treatment had an adverse effect in uninfected children (results not shown).

**Table 5:** Association between congenital toxoplasmosis and developmental outcomes: multivariable analyses

	**Developmental outcome **(Odds ratio and 95% confidence interval)			
	**PARENT COMPLETED**			
	Motor	Speech & language	Behaviour	Cognition
All centres (N = 705)	1.30 (0.75, 2.25)^2^	1.10 (0.60, 2.02) ^3,4^	1.05 (0.66, 1.67) ^2^	0.85 (0.35, 2.09)
**Sensitivity analyses**				
a) France only (N = 379)^1^	1.22 (0.61, 2.42)^2^	0.98 (0.45,2.14)	1.15 (0.64, 2.09)	0.55 (0.17, 1.76)^2^
b) Poland only (N = 134)	1.10 (0.30, 4.06)	0.54 (0.13, 2.26)	0.94 (0.30, 2.92)	1.02 (0.16, 6.62)
c) Children aged 36 to <41 months (N = 553)	1.18 (0.68, 2.05)	1.04 (0.54, 2.00)	1.25 (0.70, 2.22)	0.61 (0.25, 1.50)
d) Children with no signs before 4 months (N = 538 Poland excluded)	1.08 (0.57, 2.07)	1.10 (0.51, 2.36)	1.02 (0.57, 1.82)^2^	0.35 (0.10, 1.24)
	**CHILD COMPLETED**			
	'Draw a Man'		Line, circle, cross	
All centres (N = 705)	1.27 (0.66, 2.44)^5^		1.13 (0.56, 2.25)	
**Sensitivity analyses**				
a) France only (N = 379)^1^	0.77 (0.35, 1.67)		0.93 (0.40, 2.18)	
b) Poland only (N = 134)	1.00 (0.17, 5.77)		0.76 (0.13, 4.41)	
c) Children aged 36 to <41 months (N = 553)	0.79 (0.39, 1.63)^5^		0.96 (0.48, 1.95)	
d) Children with no signs before 4 months (N = 538 Poland excluded)	0.96 (0.46, 2.02)		1.03 (0.45, 2.36)	

All models include centre, maternal education, and child's age at completion of questionnaire

^1^adjusts for variability among centres relative to Lyon

^2^adjusted for maternal age

^3^adjusted for born outside the country

^4^adjusted for maternal age

^5^adjusted for gender, and maternal age squared

#### Parental concerns, specialist referral, and parental anxiety

Significantly more anxiety was reported in parents of infected than uninfected children. Overall the risk of having a high anxiety score was more than doubled for parents of infected children in all centers. Not surprisingly, the risk was more than trebled in Poland, where parents of infected children were compared with parents from the general population who had not been identified by screening. The association between congenital infection status and parental anxiety was attenuated and no longer significant in analyses confined to France. None of these results changed appreciably when analyses were repeated using ordinal regression. There was no evidence that duration of prenatal treatment either masked an effect of congenital infection status on parental concerns, referrals, or anxiety, or had an adverse effect on these outcomes in uninfected children (results not shown, but available from authors).

#### Parent- reported impairment

The risk of visual impairment was doubled in infected vs uninfected children (p = .024). This was mainly due to an increased risk of limited vision, affecting 7 infected and 3 uninfected children. There was no significant difference in the proportion of children wearing glasses at 3 years (6.8% infected, 4.2% uninfected; p = 0.17). Hearing impairment was more common in uninfected children but this association was not significant. The risk of neurological or mobility problems was higher in infected children, but not significant at the 5% level as this outcome was reported for only 6 children.

## Discussion

Congenital toxoplasmosis was associated with increased parental anxiety about the child's health now and in the future, and with an increased risk of visual impairment. The magnitude of the effect of congenital toxoplasmosis on parental anxiety was reduced in France. We found no evidence for an association between congenital toxoplasmosis and other markers of adverse development or behavior at three years.

These results concur with clinicians' experience, voiced during the design of the study, that parental anxiety was one of the most common problems encountered. An alternative interpretation, is that the association was a chance finding, arising because of the multiple comparisons performed. However, the consistency of the finding in sensitivity analyses, and with prior observation, make this explanation unlikely.

Other studies of children identified by screening have similarly reported that infected children appear to be developmentally normal, but no studies have systematically assessed development[2,17]. In contrast, the risk of neurological impairment is much higher in case series of referred children identified because of symptoms or abnormalities. For example, in the Chicago study, 13/45 (29%) children had an IQ/DQ score less than 85 (equivalent to one standard deviation below the mean) at 1 or 3 years old[4]. In a further case series reported by Wilson et al, 8/13 referred children had low (<50) or declining IQ/DQ scores at a mean age of 5.5 years[3].

A strength of the study is the minimization of selection bias due to referral of affected fetuses or children, by requiring that screening tests predated investigations for abnormality. As this study was prospective, data on clinical manifestations in infancy, could not be biased by the child's condition at 3 years. Conversely, we investigated whether parents that responded to the questionnaire were more or less likely to have a child with clinical manifestations detected before 4 months of age (defined as microphthalmia, microcephaly, seizures, abnormal or suspicious neurological examination requiring referral to a specialist, ventricular dilatation, or intracranial calcification) and found no significant association (reported elsewhere[7]). The association between neurological findings and abnormal developmental and impairment scores will be the subject of a further report. Nevertheless, a weakness of the study is the response rate, which was lower for uninfected than infected children.

Our analysis of reasons for non-response, found organizational attributes to be important determinants of response. Centers that provided follow up themselves, or had access to an address register, had better response rates and, apart from congenital infection status, we found no evidence of differential response rates according to other patient characteristics.[7] Uninfected children were more difficult to trace than infected children as they were discharged from follow up in late infancy, whereas infected children were followed indefinitely. However, we cannot exclude the possibility that response was associated with neurological problems more subtle than those detected in early infancy, and that the direction of this effect may have varied in infected and uninfected children.

A further weakness of the study is that we used only part of the standardized assessment tool, or a modified version of the tool, for all outcomes except behavior. This was done to enhance response by reducing the questionnaire to a reasonable length and average completion time of 21 minutes. Use of the full assessment tools, which would have required considerable time to complete, may have been more sensitive, but is unlikely to have been acceptable to parents.[18] A further compromise was to assess children at 3 years, rather than after school entry, when mild to moderate impairment may be more readily detected. Three years was decided because tracing addresses would have become increasingly difficult with age.

Our findings are consistent with the hypothesis that congenital toxoplasmosis either causes overt clinical damage, or no abnormality, rather than a spectrum of neurological impairment. However, further, more detailed assessments at older ages, including clinician assessment designed to detect mild or moderate impairment, are required to confirm or refute this hypothesis. An alternative interpretation is that the similarity in developmental outcomes in infected and uninfected children is due to anti-toxoplasma treatment. However, we found no evidence that the duration of prenatal treatment masked an adverse effect of congenital toxoplasmosis, nor that treatment caused adverse effects in uninfected children. We were unable to explore the effect of postnatal treatment in this analysis, although this will be investigated in a separate report confined to infected children.

We found an increased risk of parent-reported visual problems, which was largely due to reports of limited vision. This difference may be overestimated as parents of uninfected children were less likely to respond to this (overall response rate 92%, only 1 infected child had no response), and parents of infected children are likely to be more concerned about vision. For the seven congenitally infected children with limited vision, follow up by an ophthalmologist at 3 or more years found normal vision in 1 child, unilateral impaired vision in 5 children, and bilateral blindness in one child (results to be reported in detail elsewhere). Nevertheless, this finding concurs with a long term follow up study (median age 6 years) of 327 infected children in Lyon. None of 79 infected children with retinochoroiditis had bilateral visual impairment[19].

Generalisability of our findings should be made with caution. Our cohort was from a European setting where severely affected fetuses may be terminated. In our original cohort, there were 21 terminations: 17 were for toxoplasmosis, and 8 of these occurred after a positive prenatal diagnosis; 5 of the 8 underwent autopsy and 4 had intracranial lesions or signs of disseminated infection.[6] It is unlikely that, if these four babies had survived to three years, the results for developmental and behavioural outcomes would have been substantially altered. A further possibility is that congenital toxoplasmosis may be more severe in settings where the infecting organism load is high, as has been hypothesized for postnatal acquired infection[10]. For example, postnatal acquired ocular toxoplasmosis is both more common and more severe in Brazil, and may be associated with acquisition of infection from oocysts in unfiltered water[20]. Although a Brazilian study found a similar risk of intracranial or ocular lesions (17% of 47 infected children) to our European cohort (20%: 51/255 infected children; personal communication, R Gilbert on behalf of EMSCOT), the study was confined to women able to afford neonatal screening and may not be representative of the risk in less affluent communities[21].

Finally, the increased risk of anxiety may vary in different settings. Parents of infected children are told that eye lesions can appear at any age, and that such lesions may cause loss of vision. How this message is presented, may differ among clinicians. The effect of congenital infection status on parental anxiety was most marked in Poland, where the controls had not experienced screening, and where there is no nationwide screening program. In contrast, the risk of anxiety was lowest in France. This may be because parents are more familiar with congenital toxoplasmosis, and its limited clinical effects. Alternatively, it may be that anxiety persists in parents of uninfected children in France, thereby attenuating any difference. Marteau has reported long term effects of false positive results from other prenatal screening programs, highlighting persisting perceptions among parents that 'something must still be wrong'[9].

## Conclusion

The purpose of prenatal and neonatal screening is to reduce the risk of functional impairment. Apart from visual impairment, we found no evidence for adverse effects of congenital toxoplasmosis on developmental outcomes, behavior, or specific impairments by three years of age. On average, infected children were no worse off, and there was no evidence that this was because they received prenatal treatment. However, we could not examine the possibility that postnatal treatment ameliorated any differences in function as all infected children were treated in infancy. In addition, we cannot rule out the possibility that we failed to detect more subtle differences that would become apparent later in childhood. Finally, clinicians need to explore ways for reducing the adverse effects of diagnosis and follow up on parental anxiety. One approach is to review whether they are being sufficiently optimistic when counseling parents about prognosis.

## Competing interests

The authors declare that they have no competing interests.

## Authors' contributions

*Members of the European Multicentre Study on Congenital Toxoplasmosis (EMSCOT)

*Coordinating Committee: *M-H Bessieres, W Buffolano, H Dumon, R Gilbert, E Petersen (chairperson), A Pollak, P Thulliez, M Wallon.

*Writing Committee: *Freeman K, Salt A, Prusa A, Malm G, Ferret N, Buffolano W, Petersen E, Gilbert RE (coordinator).

*Centres contributing data *(number of patients contributed to this report): M Paul (134; University Medical Sciences, Poznan), A Prusa, M Hayde, A Pollak (134; University Children's Hospital, Vienna), M Wallon, F Peyron (134; Hôpital de la Croix Rousse, Lyon), S Romand, P Thulliez (91; Institut de Puericulture, Paris), J Franck, H Dumon, P Bastien, E Issert (69; Hôpital de la Timone, Marseille; CHU de Montpellier), W Buffolano (50; Universita di Napoli, Naples), M-H Bessieres (44; Hôpital de Rangueil, Toulouse), N Ferret, P Marty (33; Hôpital de l'Archet, Nice), H Pelloux, H Fricker-Hidalgo, C Bost-Bru (8; Centre Hospitalier Universitaire de Grenoble), G Malm, B Evengard (8; Huddinge Hospital, Stockholm), E Petersen (0; Statenseruminstitut, Copenhagen), C Chemla, (0; Hôpital Maison Blanche, Reims), E Semprini, V Savasi (0; Milan).

*Study design and coordination: *R Gilbert (Principal Investigator), L Gras, Hooi Kuan Tan, J Rickett, A Salt, L Valenti (Institute of Child Health, London)

*Statistical analysis: *Freeman K, Gras L (Institute of Child Health, London).

AS developed the questionnaires, participated in analyses and wrote the paper. KF did the statistical analyses and wrote the paper. RG had the idea for the study, obtained the funding, designed the study, developed the questionnaires, coordinated the study, and wrote the paper. All authors contributed to the design of the study and the writing of the paper. All authors read and approved the final manuscript.

**Table 6:** Association between congenital toxoplasmosis and parental concerns, specialist referral, and anxiety: multivariable analyses

	**Parental concerns and anxiety **(odds ratio and 95% confidence interval)			
	Any concerns	Any referral	Behaviour impact	Parental anxiety
All centres (N = 705)	1.22 (0.65, 2.29)^1^	0.79 (0.25, 2.55)^2^	0.57 (0.23, 1.43)	**2.59 (1.41, 4.79)**^3^
**Sensitivity analyses**				
a) France only (N = 379)	1.41 (0.73, 2.73)	0.25 (0.03, 2.09)	0.40 (0.11, 1.46)	1.63 (0.80, 3.34)
b) Poland only (N = 134)	1.06 (0.36, 3.05)	3.97 (0.90, 17.59)	0.55 (0.06, 5.40)	**3.50 (1.24, 9.84)**
c) Children aged 36 to <41 months (N = 553)	1.27 (0.73, 2.22)	1.50 (0.54, 4.13)	0.44 (0.14, 1.34)	**2.63 (1.47, 4.68)**
d) Children with no signs before 4 months (N = 538-Poland excluded)	1.00 (0.98, 1.02)	0.46 (0.10, 2.16)	0.39 (0.11, 1.38)	**2.17 (1.14, 4.15)**

All models include country, maternal education, and child's age at completion of questionnaire

^1^adjusted for gender and prenatal treatment

^2^adjusted for interaction between congenital infection status and Poland

^3^adjusted for parity

^4^adjusted for gender

## Pre-publication history

The pre-publication history for this paper can be accessed here:


